# Supplementary material for: Effect of Polyphenol Supplementation on Memory Functioning in Overweight and Obese Adults: A Systematic Review and Meta-Analysis
Source: Nutrients. 2024 Feb 6;16(4):474. doi: 10.3390/nu16040474 (PMC10893550; doi:10.3390/nu16040474)
Supplement: Supplementary file 1 [file nutrients-16-00474-s001.zip › S1 Table.pdf]

Protocol

Research Question:

Does the acute/chronic intake of polyphenols rich diet/supplementation have a positive effect on memory functions in obese/overweight population?

Inclusion criteria:

- Obese/ overweight adults of any age.
- Original article
- Peer reviewed articles in English

Research question	Does the acute/chronic intake of polyphenols rich diet/supplementation have a positive effect on memory functions in obese/overweight population??
Population	Adult age>18
Intervention	Acute and/or chronic (poly)phenols-rich supplementation
Comparator	Any: food, juice, placebo…
Outcome	Memory functions (immediate and delayed retrieval)
Study design and setting	RCT

Exclusion criteria:

- Studies written in languages other than English
- Data from congress or workshop publications
- Animal studies
- Studies in which no supplementation was given
- Studies which administered multiple supplements in addition to (poly)phenol
- Studies conducted in populations diagnosed with severe cognitive impairment/dementia
- Methodological deficiencies (e.g., allocation not randomized, absence of control comparison (e.g., Placebo (PLA) or very low poly(phenols) dose and/or content,etc.), participant not blinded, and inappropriate statistical analysis procedures),
- Case studies, encyclopedias, book chapters, and reviews were excluded, although the bibliographies of the latter were consulted to refine article searches

Data Sources and Search Strategy

Data bases: PubMed/Medline, PsycInfo, Scopus databases

Search terms:
Polyphenols component is combined with the cognition component with 'AND'

Polyphenols component: each is searched separately and then search results were combined with 'OR'	Cognition component: each is searched separately and then search results were combined with 'OR'
pomegranate or polyphenol or flavonoids, or polyphenolic compound, or polyphenolic compounds, or isoflavone, or flavanol, or phytoestrogen, or resveratrol, or ellagitannin, or ellagic acid, or punicalagin, or anthocyanins, or proanthocyanidin or proanthocyanidins.	mild cognitive impairment or MCI, or cognition, or cognitive performance, or cognitive function, or brain function, or executive function, or neuroimaging, or neural, or magnetic resonance imaging, or MRI, or fMRI, or grey matter, or gray matter, or brain structure, or electrophysiology, or EEG, or event related potential, or neuroblast, or cerebral blood flow, or CBF, or regional perfusion, or pulsatility index, or transcranial doppler, or TCD, or near-infrared spectroscopy, or NIRS, or total haemoglobin, or oxygenated haemoglobin, or oxy-Hb, or deoxygenated haemoglobin, or Deoxy-Hb.
