# Supplementary material for: Effect of Polyphenol Supplementation on Memory Functioning in Overweight and Obese Adults: A Systematic Review and Meta-Analysis
Source: Nutrients. 2024 Feb 6;16(4):474. doi: 10.3390/nu16040474 (PMC10893550; doi:10.3390/nu16040474)
Supplement: Supplementary file 1 [file nutrients-16-00474-s001.zip › S4 Table Sensitivity analysis for immediate recall.pdf]

Table S1. Sensitivity analysis of the studies (RCTs and Cross-over design) included for immediate recall meta-analysis.

**RCT**	**Model statistics with this study omitted**		
**Study omitted**	***Hedges g***	***z***	***p***
**Anton et al 2018**	**0.255**	**2.50**	**0.012**
**Boespflug et al 2017**	**0.217**	**2.05**	**0.040**
**Botwell et al 2017**	**0.243**	**2.27**	**0.023**
**Fournier et al 2007**	**0.253**	**2.33**	**0.019**
**Henderson et al 2012**	**0.249**	**2.05**	**0.040**
**Kreijkamp-Kaspers et al 2004**	**0.234**	**1.99**	**0.046**
**Rayney-Smith et al 2015**	**0.132**	**1.74**	**0.082**
**Sala-Vila et al 2020**	**0.257**	**2.23**	**0.025**
**Siddarth et al 2020**	**0.254**	**2.17**	**0.030**
**Evans et al. 2017**	**0.239**	**2.12**	**0.034**
**You et al. 2021**	**0.182**	**1.95**	**0.051**
**Herrlinger et al., 2018**	**0.201**	**1.8**	**0.061**
**Cross-over**	**Model statistics with this study omitted**		
**Alharbi et al. 2016**	**-2.385**	**-0.145**	**0.884**
**Cook et al 2020**	**3.907**	**0.251**	**0.802**
**Dodd et al 2019**	**-0.004**	**-2.523**	**0.980**
**Kennedy et al 2017**	**-0.058**	**-0.311**	**0.755**
