# Supplementary material for: Effect of Polyphenol Supplementation on Memory Functioning in Overweight and Obese Adults: A Systematic Review and Meta-Analysis
Source: Nutrients. 2024 Feb 6;16(4):474. doi: 10.3390/nu16040474 (PMC10893550; doi:10.3390/nu16040474)
Supplement: Supplementary file 1 [file nutrients-16-00474-s001.zip › S6 Table Sensitivity analysis for Delayed recall.pdf]

Table S2. Sensitivity analysis of the studies (RCTs and Cross-over design) included for delayed recall meta-analysis

RCT	Model statistics with this study omitted		
**Study omitted**	*Hedges g*	*z*	*p*
**Anton et al 2018**	**0.044**	**0.913**	**0.360**
**Botwell et al 2017**	**0.042**	**0.872**	**0.382**
**Henderson et al 2012**	**0.051**	**0.965**	**0.334**
**Huhn et al 2016**	**0.045**	**0.933**	**0.350**
**Kreijkamp-Kaspers et al 2004**	**0.024**	**0.466**	**0.641**
**Kobe et al 2017**	**0.043**	**0.894**	**0.371**
**Rayney-Smith et al 2015**	**0.031**	**0.621**	**0.534**
**Sala-Vila et al 2020**	**0.077**	**1.261**	**0.207**
**Siddarth et al 2020**	**0.035**	**0.682**	**0.494**
**Witte et al 2014**	**0.032**	**0.652**	**0.514**
**Evans et al. 2017**	**0.031**	**0.638**	**0.523**
**Krikorian et al. 2022**	**0.049**	**1.003**	**0.315**
**Cross-over**	**Model statistics with this study omitted**		
**Alharbi et al. 2016**	**-0.114**	**-0.789**	**0.430**
**Kennedy et al 2017**	**-0.116**	**-0.817**	**0.413**
**Dodd et al 2019**	**-0.153**	**-0.950**	**0.341**
**Whyte et al. 2021**	**-0.070**	**-0.445**	**0.655**
