# Supplementary material for: Effect of Polyphenol Supplementation on Memory Functioning in Overweight and Obese Adults: A Systematic Review and Meta-Analysis
Source: Nutrients. 2024 Feb 6;16(4):474. doi: 10.3390/nu16040474 (PMC10893550; doi:10.3390/nu16040474)
Supplement: Supplementary file 1 [file nutrients-16-00474-s001.zip › S7 Raw data for immediate recall metaanalysis.pdf]

	Study name	Comparison
1		
2		
3		
4		
5		
6		
7		
8		
9		
10		
11		
12		
13		
14		
15		
16		
17		
18		
19		
20		
21		
22		
23		
24		
25		
26		
27		
28		
29		
30		
31		
32		
33		
34		
35		
36		
37		
38		
39		

	Data format	Intervention Mean	Intervention Std-Dev	Intervention Sample size	Control Mean	Control Std-Dev	Control Sample size	Effect direction	Std diff in means	Std Err
1									0.000	0.302
2									0.094	0.302
3									-0.221	0.449
4									-0.832	0.446
5									1.342	0.553
6									0.199	0.501
7									0.000	0.500
8									0.137	0.394
9									-0.286	0.395
10									-0.599	0.386
11									-0.110	0.378
12									-0.125	0.378
13									-0.301	0.335
14									0.092	0.334
15									0.590	0.341
16									0.198	0.334
17									-0.291	0.335
18									-0.465	0.338
19									-0.015	0.333
20									0.013	0.333
21									-0.392	0.280
22									-0.194	0.275
23									0.000	0.278
24									-0.065	0.275
25									-0.118	0.278
26									0.117	0.275
27									-0.110	0.278
28									0.016	0.275
29									-0.078	0.113
30									0.156	0.113
31									0.256	0.114
32									0.261	0.152
33									0.087	0.151
34									0.261	0.152
35									-0.074	0.218
36									0.134	0.218
37									0.029	0.218
38									0.363	0.220
39									-0.282	0.219

	Hedges's g	Std Err	Difference in means	Std Err	Duration of Diet	K
1	0.000	0.296	0.000	0.583		
2	0.092	0.296	0.200	0.640		
3	-0.211	0.430	-0.630	1.277		
4	-0.800	0.429	-2.070	1.066		
5	1.268	0.523	0.030	0.011		
6	0.188	0.474	0.020	0.050		
7	0.000	0.473	0.000	0.043		
8	0.133	0.381	0.015	0.043		
9	-0.277	0.383	-0.052	0.071		
10	-0.582	0.375	-10.000	6.308		
11	-0.107	0.367	-1.000	3.423		
12	-0.121	0.367	-1.000	3.024		
13	-0.294	0.328	-0.600	0.665		
14	0.090	0.326	0.180	0.651		
15	0.577	0.333	1.290	0.728		
16	0.194	0.327	0.500	0.842		
17	-0.284	0.328	-0.530	0.608		
18	-0.455	0.330	-0.750	0.537		
19	-0.015	0.326	-0.290	6.350		
20	0.013	0.326	0.250	6.251		
21	-0.386	0.276	-0.800	0.567		
22	-0.191	0.271	-0.400	0.566		
23	0.000	0.273	0.000	0.425		
24	-0.064	0.271	-0.100	0.424		
25	-0.116	0.274	-0.300	0.708		
26	0.115	0.271	0.300	0.707		
27	-0.108	0.274	-1.400	3.541		
28	0.015	0.271	0.200	3.537		
29	-0.078	0.113				
30	0.155	0.113				
31	0.256	0.113				
32	0.259	0.151	0.300	0.174		
33	0.086	0.151	0.100	0.174		
34	0.260	0.151	2.600	1.507		
35	-0.074	0.216	-0.630	1.846		
36	0.133	0.216	1.150	1.874		
37	0.029	0.216	0.240	1.819		
38	0.359	0.218	3.150	1.895		
39	-0.280	0.217	-2.400	1.855		

	Study name	Comparison
40		
41		
42		
43		
44		
45		
46		
47		
48		
49		
50		
51		
52		
53		
54		
55		
56		
57		
58		
59		
60		
61		
62		
63		
64		
65		
66		
67		
68		
69		
70		
71		
72		
73		
74		
75		
76		
77		
78		

	Data format	Intervention Mean	Intervention Std-Dev	Intervention Sample size	Control Mean	Control Std-Dev	Control Sample size	Effect direction	Std diff in means	Std Err
40									0.243	0.219
41									-0.108	0.218
42									0.009	0.218
43									-0.440	0.221
44									-0.021	0.218
45									0.070	0.218
46									-0.103	0.218
47									0.196	0.219
48									0.170	0.219
49									-0.040	0.218
50									0.431	0.221
51									0.158	0.230
52									0.128	0.230
53									0.082	0.230
54									0.180	0.230
55									-0.036	0.229
56									0.067	0.229
57									0.168	0.230
58									-0.052	0.229
59									0.288	0.231
60									-0.020	0.229
61									-0.028	0.229
62									-0.216	0.230
63									-0.030	0.229
64									-0.050	0.229
65									0.069	0.229
66									0.222	0.230
67									0.154	0.219
68									0.360	0.220
69									0.107	0.218
70									0.053	0.218
71									-0.028	0.218
72									0.021	0.218
73									0.136	0.218
74									0.124	0.218
75									0.073	0.218
76									-0.064	0.218
77									0.261	0.219
78									0.086	0.218

	Hedges's g	Std Err	Difference in means	Std Err	Duration of Diet	K
40	0.240	0.217	2.130	1.916		
41	-0.107	0.216	-0.910	1.842		
42	0.008	0.216	0.070	1.794		
43	-0.436	0.219	-3.380	1.677		
44	-0.021	0.216	-0.170	1.791		
45	0.069	0.216	0.560	1.747		
46	-0.102	0.216	-0.830	1.761		
47	0.194	0.217	1.760	1.961		
48	0.168	0.217	1.330	1.711		
49	-0.040	0.216	-0.370	2.002		
50	0.427	0.219	3.420	1.732		
51	0.156	0.227	1.890	2.748		
52	0.127	0.227	1.490	2.662		
53	0.081	0.227	1.080	3.010		
54	0.178	0.228	2.120	2.707		
55	-0.036	0.227	-0.470	3.003		
56	0.066	0.227	0.890	3.044		
57	0.166	0.227	1.870	2.555		
58	-0.051	0.227	-0.680	3.010		
59	0.285	0.228	3.290	2.622		
60	-0.020	0.227	-0.240	2.741		
61	-0.028	0.227	-0.330	2.701		
62	-0.214	0.228	-2.620	2.781		
63	-0.030	0.227	-0.350	2.673		
64	-0.049	0.227	-0.590	2.709		
65	0.068	0.227	0.810	2.701		
66	0.220	0.228	2.680	2.767		
67	0.153	0.217	0.470	0.665		
68	0.357	0.218	1.160	0.703		
69	0.106	0.216	0.320	0.652		
70	0.053	0.216	0.170	0.697		
71	-0.028	0.216	-0.090	0.697		
72	0.021	0.216	0.070	0.715		
73	0.135	0.216	0.420	0.672		
74	0.123	0.216	0.370	0.651		
75	0.073	0.216	0.240	0.714		
76	-0.064	0.216	-0.210	0.715		
77	0.258	0.217	0.760	0.636		
78	0.086	0.216	0.280	0.708		

	Study name	Comparison
79		
80		
81		
82		
83		
84		
85		
86		
87		
88		
89		
90		
91		
92		
93		
94		
95		
96		

	Data format	Intervention Mean	Intervention Std-Dev	Intervention Sample size	Control Mean	Control Std-Dev	Control Sample size	Effect direction	Std diff in means	Std Err
79									0.415	0.221
80									0.006	0.218
81									-0.072	0.218
82									-0.107	0.218
83									0.000	0.208
84									2.035	0.254
85									-0.031	0.193
86									0.296	0.194
87									-0.105	0.193
88									0.077	0.193
89									-0.079	0.078
90									-0.034	0.078
91									0.017	0.078
92									0.026	0.078
93									0.018	0.141
94									0.189	0.139
95									-0.007	0.141
96									0.021	0.139

	Hedges's g	Std Err	Difference in means	Std Err	Duration of Diet	K
79	0.411	0.219	1.210	0.636		
80	0.006	0.216	0.020	0.707		
81	-0.071	0.216	-0.200	0.609		
82	-0.106	0.216	-0.360	0.737		
83	0.000	0.206	0.000	0.071		
84	2.019	0.252	0.700	0.071		
85	-0.031	0.192	-0.010	0.063		
86	0.294	0.193	0.195	0.127		
87	-0.104	0.192	-0.091	0.167		
88	0.077	0.192	0.061	0.152		
89	-0.079	0.078	-0.050	0.049		
90	-0.034	0.078	-0.370	0.849		
91	0.017	0.078	0.340	1.583		
92	0.026	0.078	0.740	2.256		
93	0.018	0.141	0.100	0.783		
94	0.188	0.139	1.000	0.737		
95	-0.007	0.141	-0.100	2.030		
96	0.021	0.139	0.300	2.016		
