# Supplementary material for: Effect of Polyphenol Supplementation on Memory Functioning in Overweight and Obese Adults: A Systematic Review and Meta-Analysis
Source: Nutrients. 2024 Feb 6;16(4):474. doi: 10.3390/nu16040474 (PMC10893550; doi:10.3390/nu16040474)
Supplement: Supplementary file 1 [file nutrients-16-00474-s001.zip › S8 Raw data for delayed recall metaanalysis.pdf]

	Study name	Comparison
1		
2		
3		
4		
5		
6		
7		
8		
9		
10		
11		
12		
13		
14		
15		
16		
17		
18		
19		
20		
21		
22		
23		
24		
25		
26		
27		
28		
29		
30		
31		
32		
33		
34		
35		
36		
37		
38		
39		

	Data format	Intervention Mean	Intervention Std-Dev	Intervention Sample size	Control Mean	Control Std-Dev	Control Sample size	Effect direction	Std diff in means	Std Err
1									-0.244	0.303
2									0.027	0.302
3									-0.739	0.442
4									-0.600	0.457
5									-0.040	0.447
6									0.522	0.435
7									-0.415	0.398
8									-0.113	0.394
9									0.416	0.398
10									-0.189	0.334
11									-0.144	0.334
12									0.253	0.335
13									-0.313	0.335
14									0.034	0.115
15									0.113	0.114
16									-0.163	0.113
17									-0.219	0.276
18									-0.255	0.276
19									0.173	0.275
20									0.197	0.152
21									-0.056	0.218
22									-0.256	0.219
23									-0.045	0.218
24									0.032	0.218
25									-0.029	0.218
26									-0.004	0.218
27									-0.217	0.219
28									0.024	0.218
29									0.061	0.218
30									0.436	0.221
31									-0.301	0.219
32									0.316	0.220
33									0.008	0.218
34									0.139	0.218
35									-0.106	0.218
36									0.074	0.218
37									-0.178	0.227
38									0.097	0.227
39									-0.271	0.227

	Hedges's g	Std Err	Difference in means	Std Err	Duration of diet	R
1	-0.239	0.297	-0.800	0.990		
2	0.026	0.296	0.100	1.131		
3	-0.711	0.426	-1.670	0.967		
4	-0.575	0.438	-1.500	1.118		
5	-0.038	0.428	-0.300	3.369		
6	0.502	0.419	6.230	5.110		
7	-0.402	0.385	-0.900	0.854		
8	-0.110	0.381	-0.030	0.104		
9	0.403	0.385	1.500	1.419		
10	-0.185	0.327	-0.450	0.792		
11	-0.141	0.326	-0.410	0.948		
12	0.247	0.327	0.940	1.238		
13	-0.306	0.328	-1.380	1.471		
14	0.033	0.114				
15	0.113	0.113				
16	-0.163	0.113				
17	-0.216	0.272	-0.670	0.840		
18	-0.252	0.272	-0.680	0.732		
19	0.171	0.271	2.875	4.561		
20	0.196	0.151	0.600	0.461		
21	-0.055	0.216	-0.880	3.451		
22	-0.254	0.217	-4.230	3.601		
23	-0.044	0.216	-0.770	3.755		
24	0.032	0.216	0.570	3.897		
25	-0.029	0.216	-0.480	3.557		
26	-0.004	0.216	-0.070	3.476		
27	-0.215	0.217	-3.350	3.369		
28	0.024	0.216	0.370	3.319		
29	0.060	0.216	0.910	3.275		
30	0.432	0.219	6.480	3.245		
31	-0.298	0.217	-4.340	3.148		
32	0.313	0.218	4.730	3.267		
33	0.008	0.216	0.120	3.247		
34	0.138	0.216	2.110	3.309		
35	-0.105	0.216	-1.550	3.199		
36	0.073	0.216	1.160	3.424		
37	-0.176	0.225	-1.090	1.387		
38	0.096	0.224	0.590	1.372		
39	-0.268	0.225	-1.760	1.471		

	Study name	Comparison
40		
41		
42		
43		
44		
45		
46		
47		
48		
49		
50		
51		
52		
53		
54		
55		
56		
57		
58		
59		
60		
61		
62		
63		
64		
65		
66		
67		
68		
69		
70		
71		
72		
73		
74		
75		
76		
77		
78		

	Data format	Intervention Mean	Intervention Std-Dev	Intervention Sample size	Control Mean	Control Std-Dev	Control Sample size	Effect direction	Std diff in means	Std Err
40									-0.219	0.227
41									-0.096	0.227
42									0.387	0.229
43									-0.036	0.226
44									0.145	0.227
45									-0.116	0.227
46									0.284	0.228
47									-0.006	0.226
48									-0.120	0.227
49									-0.068	0.227
50									-0.086	0.227
51									-0.135	0.227
52									-0.118	0.227
53									-0.170	0.230
54									-0.233	0.230
55									0.190	0.230
56									-0.245	0.230
57									-0.133	0.230
58									-0.142	0.230
59									0.020	0.229
60									-0.053	0.229
61									-0.099	0.230
62									-0.133	0.230
63									0.069	0.229
64									-0.076	0.229
65									-0.063	0.229
66									-0.457	0.232
67									-0.286	0.231
68									-0.295	0.231
69									-0.148	0.224
70									0.189	0.224
71									-0.141	0.224
72									-0.313	0.225
73									0.029	0.224
74									0.047	0.224
75									-0.164	0.224
76									0.078	0.224
77									0.299	0.225
78									0.064	0.224

	Hedges's g	Std Err	Difference in means	Std Err	Duration of diet	R
40	-0.217	0.225	-1.510	1.560		
41	-0.095	0.224	-0.640	1.503		
42	0.383	0.226	2.260	1.322		
43	-0.035	0.224	-0.230	1.456		
44	0.144	0.225	1.090	1.697		
45	-0.115	0.224	-0.770	1.500		
46	0.282	0.225	1.950	1.552		
47	-0.006	0.224	-0.040	1.408		
48	-0.119	0.224	-0.840	1.588		
49	-0.068	0.224	-0.430	1.423		
50	-0.085	0.224	-0.610	1.611		
51	-0.134	0.224	-0.870	1.454		
52	-0.117	0.224	-0.810	1.550		
53	-0.168	0.227	-1.850	2.499		
54	-0.231	0.228	-2.780	2.732		
55	0.188	0.228	2.090	2.530		
56	-0.243	0.228	-3.090	2.892		
57	-0.132	0.227	-1.680	2.901		
58	-0.140	0.227	-1.640	2.651		
59	0.020	0.227	0.240	2.743		
60	-0.052	0.227	-0.630	2.752		
61	-0.098	0.227	-1.150	2.671		
62	-0.132	0.227	-1.460	2.511		
63	0.068	0.227	0.770	2.568		
64	-0.076	0.227	-0.890	2.674		
65	-0.063	0.227	-0.700	2.538		
66	-0.452	0.230	-5.140	2.581		
67	-0.283	0.228	-2.820	2.264		
68	-0.292	0.228	-3.170	2.467		
69	-0.147	0.222	-1.270	1.914		
70	0.187	0.222	1.670	1.973		
71	-0.140	0.222	-1.260	1.995		
72	-0.310	0.223	-2.600	1.856		
73	0.029	0.221	0.250	1.933		
74	0.047	0.221	0.390	1.854		
75	-0.162	0.222	-1.390	1.895		
76	0.077	0.222	0.670	1.917		
77	0.296	0.223	2.460	1.839		
78	0.063	0.222	0.560	1.969		

	Study name	Comparison
79		
80		
81		
82		
83		
84		
85		
86		
87		
88		
89		
90		
91		
92		
93		
94		
95		

	Data format	Intervention Mean	Intervention Std-Dev	Intervention Sample size	Control Mean	Control Std-Dev	Control Sample size	Effect direction	Std diff in means	Std Err
79									0.080	0.224
80									-0.068	0.224
81									0.040	0.224
82									0.137	0.224
83									0.135	0.224
84									-0.075	0.224
85									-0.237	0.319
86									0.115	0.318
87									0.000	0.208
88									0.451	0.210
89									-0.018	0.078
90									0.189	0.142
91									0.205	0.139
92									-0.045	0.141
93									0.000	0.139
94									0.515	0.300
95									0.252	0.296

	Hedges's g	Std Err	Difference in means	Std Err	Duration of diet	R
79	0.080	0.222	0.650	1.810		
80	-0.067	0.222	-0.620	2.042		
81	0.040	0.221	0.340	1.884		
82	0.135	0.222	1.180	1.930		
83	0.134	0.222	1.070	1.768		
84	-0.074	0.222	-0.660	1.967		
85	-0.233	0.313	-0.900	1.205		
86	0.113	0.312	0.700	1.936		
87	0.000	0.206	0.000	0.092		
88	0.448	0.209	0.200	0.092		
89	-0.018	0.078	-0.160	0.699		
90	0.189	0.141	0.400	0.299		
91	0.204	0.139	0.400	0.271		
92	-0.045	0.141	-0.100	0.312		
93	0.000	0.139	0.000	0.278		
94	0.506	0.295	1.700	0.973		
95	0.248	0.291	0.500	0.584		
