# Supplementary figures and images for: Effect of Polyphenol Supplementation on Memory Functioning in Overweight and Obese Adults: A Systematic Review and Meta-Analysis
Source: Nutrients. 2024 Feb 6;16(4):474. doi: 10.3390/nu16040474 (PMC10893550; doi:10.3390/nu16040474)

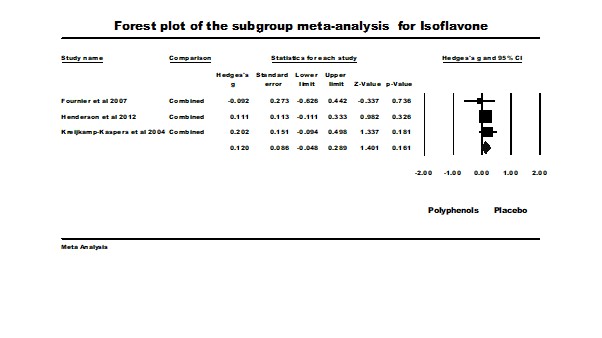

Supplement: Supplementary file 1 [file nutrients-16-00474-s001.zip › S2 Figure Sub-group meta-analysis for isoflavone.jpg]

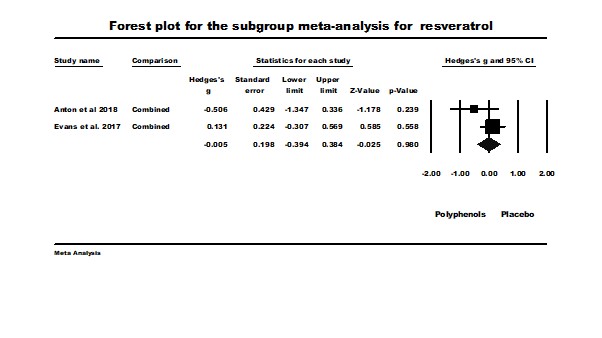

Supplement: Supplementary file 1 [file nutrients-16-00474-s001.zip › S3 Figure Sub-group meta-analysis for Resveratrol.jpg]

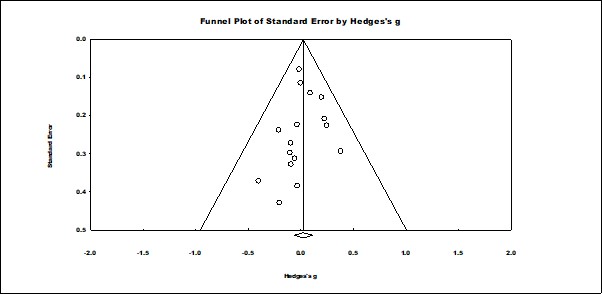

Supplement: Supplementary file 1 [file nutrients-16-00474-s001.zip › S5 Figure Funnel plot for delayed retrival.jpg]
